# Wetting Transitions of Liquid Gallium Film on Nanopillar-Decorated Graphene Surfaces

**DOI:** 10.3390/molecules23102407

**Published:** 2018-09-20

**Authors:** Junjun Wang, Tao Li, Yifan Li, Yunrui Duan, Yanyan Jiang, Hamidreza Arandiyan, Hui Li

**Affiliations:** 1 Key Laboratory for Liquid-Solid Structural Evolution and Processing of Materials, Ministry of Education, Shandong University, Jinan 250061, China; wangjunjun_1019@hotmail.com (J.W.); lt1126322737@hotmail.com (T.L.); liyifan12181123@hotmail.com (Y.L.); yrduan@outlook.com (Y.D.); 2 Department of Physics, Changji University, Changji 831100, China; 3 Laboratory of Advanced Catalysis for Sustainability, School of Chemistry, The University of Sydney, Sydney 2006, Australia

**Keywords:** wetting transition, liquid gallium, nanopillar-decorated graphene surface, adhesion energy

## Abstract

Molecular dynamics (MD) simulation has been employed to study the wetting transitions of liquid gallium droplet on the graphene surfaces, which are decorated with three types of carbon nanopillars, and to explore the effect of the surface roughness and morphology on the wettability of liquid Ga. The simulation results showed that, at the beginning, the Ga film looks like an upside-down dish on the rough surface, different from that on the smooth graphene surface, and its size is crucial to the final state of liquid. Ga droplets exhibit a Cassie–Baxter (CB) state, a Wenzel state, a Mixed Wetting state, and a dewetting state on the patterned surfaces by changing distribution and the morphology of nanopillars. Top morphology of nanopillars has a direct impact on the wetting transition of liquid Ga. There are three transition states for the two types of carbon nanotube (CNT) substrates and two for the carbon nanocone (CNC) one. Furthermore, we have found that the substrates show high or low adhesion to the Ga droplet with the variation of their roughness and top morphology. With the roughness decreasing, the adhesion energy of the substrate decreases. With the same roughness, the CNC/graphene surface has the lowest adhesion energy, followed by CNT/graphene and capped CNT/graphene surfaces. Our findings provide not only valid support to previous works but also reveal new theories on the wetting model of the metal droplet on the rough substrates.

## 1. Introduction

The stretchable electronics achieve remarkable progress in soft robotics [1,2,3], flexible devices [4,5,6,7,8], and especially the biological field [5,9]. Room temperature liquid metals have drawn increasing attention in state-of-the-art applications in these fields because the desirable materials involved are intrinsically soft and remain functionally stable when their morphology changes [1,6,7,9]. Gallium and their alloys, being in a liquid state at room temperature with high thermal and electrical conductivity, with low toxicity and evaporation pressure, make them ideal candidates for a myriad of applications. Gallium is regarded as a promising alternative to the toxic mercury. Graphene, with controllable stiffness performances, high electrical conductivity, and a low synthesis cost, also possesses desirable deformability, which can significantly support the gallium-based liquid metal as an electrically conductive and anticorrosive coating [10,11,12]. The effective combination of these two typical classes of materials has attracted considerable interest. On the one hand, the flexibility gives them the capability of following tortuous paths with little interaction with the working environment and with little risk of disrupting other devices. Ordonez et al. reported a flexible device that 2-D graphene combined with Galinstan (consisting of 68.5% Ga, 21.5% In, and 10% Sn), exhibited a resistance chance of less than 5.5% when subjected to repeating deformation with a radius of curvature as small as 4.5 mm [7]. On the other hand, controllable rigidity devices are required under external interventions to transmit signals, for example in medical examination, for responding to other tools and upgrading precision in positioning [5]. However, the low viscoelastic and excellent wetting characteristics of gallium and their alloys have become a major obstacle, restricting those available liquid metals from promising applications. In consequence, effectively controlling the wetting properties of liquid metals is crucial to the manufacture of stretchable electronics containing liquid metal.

Some external assistance, such as external pressure [13] or force [14], electric [15,16] or magnetic fields [17], mechanical vibrations [18,19], and changing temperature [20], has been employed to control the wettability of liquid metals. However, these strategies have loads of demerits. For instance, temperature variation and current pulses generate additional heat input [15,16,20], which may be harmful to an organism. Furthermore, the mechanical vibration is difficult to implement in a microdevice, and the magnetic field has unique requirements for the application [17]. In consideration of such limitations, how to control the wetting transitions of liquid metals on a substrate remains a significant challenge. At present, wetting transitions of liquid droplets on the substrate have gained notable achievements, and two parameters are mainly referred: the surface energy of the materials and the surface roughness [21,22,23,24,25,26,27,28,29,30].

**Figure 1:**
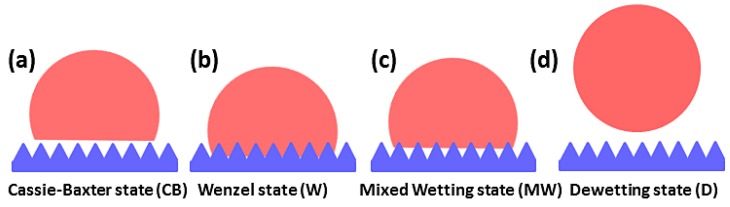
Schematic of the wetting states: (**a**) the Cassie–Baxter (CB) model; (**b**) the Wenzel (W) model, (**c**) the Mixed Wetting (MW) model; and (**d**) the dewetting (D) model.

There are various models proposed to explain different wetting states (Figure 1). Generally, the wetting states contain four different cases: a Cassie–Baxter (CB) state, a Wenzel (W) state, a Mixed Wetting (MW) state (a state between CB and W state), and a dewetting (D) state. In the CB case (Figure 1a), the contact angle *θ_CA_* is supplied by the following equation [31]:

(1) $$\;\cos\theta_{CA}=-1+f_{c}\left(\cos\theta_{s}+1\right)\;$$

where *f_c_* is the relative area fraction of the patterned solid and surfaces underneath the droplet, and *θ_s_* is the contact angle of liquid on smooth surfaces.

In the W case (as shown in Figure 1b), the contact angle *θ_CA_* is yielded as [32]

(2) $$\;\cos\theta_{CA}=r\cos\theta_{s}\;$$

where *r* is the roughness ratio of the substrate.

In the MW case (as shown in Figure 1c), the contact angle *θ_CA_* is given by [27,29]

(3) $$\;\cos\theta_{CA}=1-f_{c}+f_{c}\cos\theta_{s}\;$$

However, the underlying mechanism, particularly regarding the relationship among the wettability, the top morphology of nanopillars, and the surface roughness, is not fundamentally understood. We performed MD simulations to investigate the wetting transition of a pancake-like Ga nanofilm on three types of carbon nanopillar-patterned graphene surfaces, with the aim of controlling the wetting pattern of liquid Ga and exploring the general rule on its wettability on the graphene-based substrate.

## 2. Methods and Models

**Figure 2:**
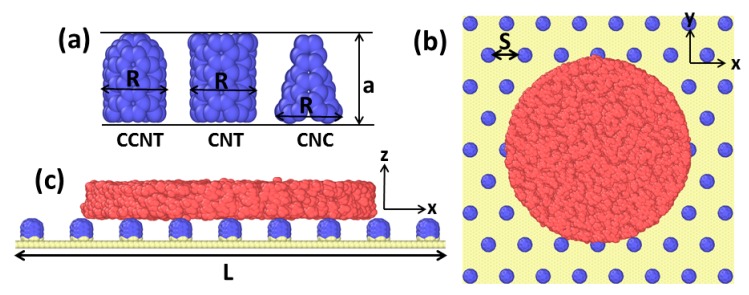
Initial simulation model. Ga atoms are shown in peachy beige, carbon nanopillars in blue, and graphene in yellow. (**a**) Initial atomic configuration for three kinds of carbon nanopillars; (**b**) the top view and (**c**) the side view of the whole system at *t* = 0 ps.

Molecular dynamics (MD) simulations were employed using the “Large-scale Atomic/Molecular Massively Parallel Simulator” (LAMMPS) [33]. The gallium pancake-like film with a height of 20 Å and a diameter of 135.6 Å was placed on the rough surface with horizontal dimensions of 200 × 200 Å^2^, regularly decorated with carbon nanopillars which are made of carbon nanotubes (CNTs) (5, 5), caped carbon nanotubes (CCNTs) (5, 5), and carbon nanocones (CNCs, with a disclination angle of 240°), a constant height of 10 Å, and a bottom diameter of 6.78 Å, respectively. In order to study the effect of the substrate roughness on Ga droplet wettability, six sets of interpillar spacing (side to side) were used in this study. The distance between any two nearest neighbor nanopillars on the substrate was set as six values of 3.4 Å, 7 Å, 10.5 Å, 14 Å, 17.5 Å, and 20 Å, where 3.4 Å is the theoretical minimum distance. The suitable vertical distance between the Ga film and the top of the substrate was 2 Å. Figure 2 shows the parameters of the pattern on the substrates: the radius of a nanopillar *R*, the distance between each two nanopillars *S*, the height of a nanopillar *a*, the length side of rough substrate *L*, and the number of nanopillars patterned on the same basal area of the substrate *n*. The roughness ratio of substrate *r* and area fraction *f_c_* was calculated using the following formulas [26] (the values adopted in our calculation is as shown in Table 1):

(4) $$\;r=1+\frac{1}{2}n\pi^{2}aRL^{-2}\;$$

(5) $$\;f_{c}=n\pi R^{2}L^{-2}.\;$$

In addition, the adhesion energy per unit area *E_A_*, which describes the energy needed to separate liquid from the solid surface, was also used [34,35,36] and is given by the following equation:

(6) $$\;E_{A}=-\left[E_{total}-\left(E_{surface}+E_{liquid}\right)\right]A^{-1}\;$$

where *E_total_* is the potential energy of the total equilibrated system, *E_surface_* is the potential energy of the solid surface, *E_liquid_* is the potential energy of the isolated Ga film, and *A* is the cross-section area of the model from the *z*-axis.

In our simulations, the periodic boundary conditions were used in all three spatial dimensions. The interaction of Ga atoms was governed by the modified embedded atom method (MEAM) potential [37,38]. The interaction of carbon atoms was employed by the adaptive intermolecular reactive empirical bond order (AIREBO) potential [39]. The interaction between Ga and C was modeled with a 12–6 Lennard–Jones potential with a well depth *ε* of 0.005 eV, a size parameter *σ* of 3.18 Å, and a cutoff of 10.0 Å [40,41]. The Ga–graphite system has mainly deduced this LJ potential with an equilibrium contact angle *θ_CA_* of 129.6°, which is an average measured value similar to the experimental results of *θ_CA_* = 127° [42]. The time step was valued at 1 fs in all cases. The simulation courses were carried out in two stages: firstly, Ga atoms were equilibrated with Berendsen thermostat in an NPT ensemble until liquid Ga reached a stable state, and secondly, the NVT ensemble was applied for the Ga droplet on each kind of substrate sustaining 1000 K controlled by the Nose–Hoover method [43]. The original box had dimensions of 310 × 310 × 310 Å. The graphene was initially present within 20 Å from the wall of the box.

Thus, the interaction between C and Ga atoms enabled liquid Ga to be lifted off from the solid substrate. However, it is important to note that this process was controlled at a constant cross-sectional area of the solid substrate, a constant pressure, a constant temperature, and a constant number of particles. Therefore, the Gibbs free energy change will be discussed.

## 3. Results and Discussion

### 3.1. Summary of Theoretical Results

The theoretical results of the Ga droplet on 18 different substrates with different roughness (the distance *S* between each two nanopillars) and top morphology surfaces (CCNT, CNT, and CNC) is listed in Table 1. In Table 1, the surface roughness (*r*), the area fraction (*f_c_*), the apparent contact angle (*θ_CA_*), the theoretical contact angle (*θ_TCA_* derived from Equations (1)–(3), and the wetting state are given. For CNT/G (G is the abbreviation of graphene) substrates, with the increase in *S*, it undergoes a transition from the CB state to the D state and the contact angle *θ_CA_* also rises because the smaller gaps restrict the Ga atoms to move up among the nanopillars. Interestingly, the wetting state of the Ga droplet on CCNT/G substrates gradually shows the CB state, MW state, and D state as the *S* increases. In the CB state, the apparent contact angle *θ_CA_* on CCNT/G is slightly smaller than that on CNT/G. However, as for the Ga droplet on the CNC/G substrate, the reverse wetting transition process was observed. With *S* increasing, the Ga droplet on the CNC substrate firstly experiences the D state and then the W state, where *θ_CA_* decreases. In addition, for the Ga droplet on the CNT/G and CNC/G substrates, the difference between *θ_CA_* and *θ_TCA_* is relatively small compared with that on the CCNT/G substrate. Primarily, the apparent contact angle *θ_CA_* in the MW state on the CCNT/G is far from the theoretical result and even closer to the value of the CB model. This implies that the immersion depth of liquid atoms to the rough surface is small enough, which makes it more fit for the CB model instead of the MW model. Nevertheless, to distinguish the wetting states of CCNT7/G (*S* = 7 Å) and CCNT10/G (*S* = 10.5 Å) to others, both cases are still called the MW state in this article. In addition, Table 1 demonstrates that, for CNT/G and CCNT/G substrates, a small *S* value can strengthen the hydrophilicity, but this is not the same for the CNC/G substrates.

### 3.2. The Effect of Topography on Initial Wetting States

**Figure 3:**
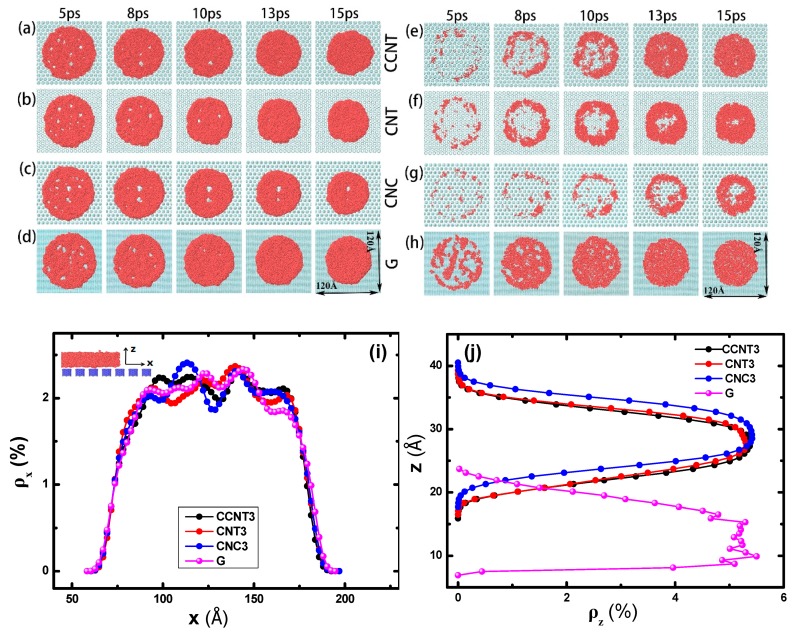
The effect of the nanopillar top on the wetting properties of Ga droplet bottoms on substrates. The wetting scenario in the first 15 ps of Ga droplets on (**a**) CCNT3/G, (**b**) CNT3/G, (**c**) CNC3/G, and (**d**) G (graphene) substrates. The bottom of Ga atoms within 3 Å distance to the following substrates: (**e**) CCNT3/G, (**f**) CNT3/G, (**g**) CNC3/G, and (**h**) G substrates. The Ga number density *ρ_nx_* of along the (**i**) *x*- and (**j**) *z*-directions at 8 ps.

The morphology of the Ga film on different substrates was investigated. Figure 3a–d show the wetting pattern of the Ga films on different substrates during the first 15 ps. In the initial stage, the Ga film begins to fracture and form some small holes, and the holes then gradually fade, which corresponds to both experimental and simulated results [44,45]. Compared with the smooth graphene, the modified graphene surface slows down the fading of holes because the rough surface retards the movement of Ga atoms.

To further understand the core structure of liquid Ga on each substrate, we also compared the density profiles of the liquid Ga film on three rough surfaces and one smooth surface at 8 ps in Figure 3i,j. The number density *ρ_ni_* (*i* = *x*, *y* or *z*) is a structural parameter of the density distribution in one direction, which can measure the numbers of certain particles in the space. Here,

(7)  ρni=niNliq 

where *n_i_* and *N_liq_* are the numbers of Ga atoms in small volume (e.g., *i* = *x*, d*v* = *y z* d*x*) and the entire volume, respectively. Figure 3i illustrates that, at 8 ps, the diameters of Ga films on three rough surfaces are almost identical to the smooth one, but the distribution rules of Ga atoms at the central areas in the horizontal direction exhibit a height difference. For example, on the CNC3/G (*S* = 3.4 Å) substrate, near the center (*x* = 130 Å) region, the *ρ_nx_* of Ga atoms has one evident minimum, while several local minimal values appear at the center of the graphene surface. It is shown in Figure 3j that the mass center of the Ga film on the rough surface locates in the middle layer in the *z*-direction but in the underlayer on the smooth surface, which indicates that the rough surface increases the repellency or decreases absorbency between the liquid and solid and slows down the sinking of Ga atoms. Furthermore, for the patterned surface, the number density distribution of Ga atoms is extraordinarily distinct where it shows an approximately normal distribution along the *z*-direction. However, Ga atoms on the graphene surface are scattered randomly, especially in the middle section.

We sliced the liquid films to investigate the inner structure of the Ga film. After removing the Ga atoms at a distance of 3 Å above the substrate in the first 15 ps (shown in Figure 3e–g), it can be seen that the rest of Ga film shows a large hole in the center on the rough surfaces, while several nonuniform small holes are found to distribute randomly on the smooth graphene (Figure 3h). This means that, at the beginning, the rough substrate makes the Ga film looks like an upside-down dish, corresponding to the distribution of the number density. The dish shows different diameters and depths because of the different morphology of the nanopillars, and for each substrate the diameter appears to scale with the depth. On the CNC3/G substrate, the dish diameter (or depth) is the largest, that on the CNT3/G substrate the second largest, and that on the CCNT3/G substrate the smallest. As shown in Table 1, on CCNT3/G and CNT3/G substrates, Ga droplets remain stable at the CB wetting state but the contact angle of CNT3/G is slightly larger. The larger the inner size of dish is, the lower the strength of the surface adsorption is. This indicates that the patterned surface reduces its contact with the liquid film in the primary stage and that the CNC nanopillars can make the graphene surface more hydrophobic than the CCNT and CNT ones.

### 3.3. The Effect of Topography on Wetting Transition

**Figure 4:**
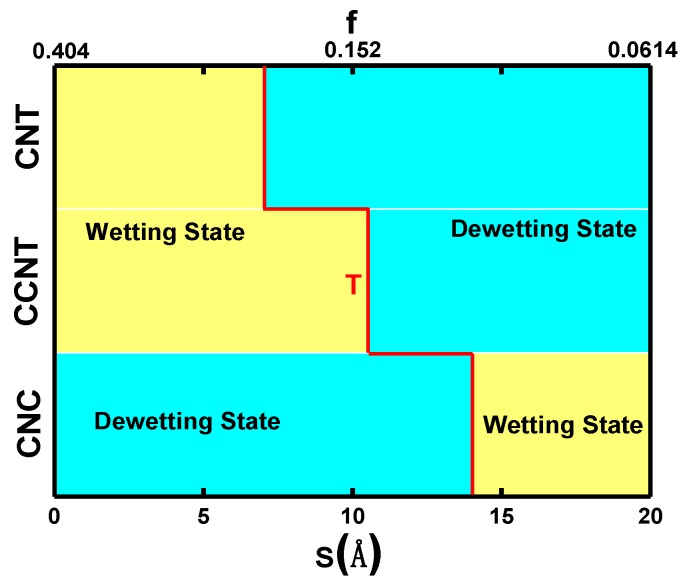
Wetting transition between the wetting state and the dewetting state in all surfaces, where transition line T is red.

Figure 4 summarizes the wetting transition graph on three kinds of decorated substrates, in which wetting states are divided by a colored transition line T. According to Equations (4) and (5), a smaller S results in a higher surface roughness. For the CNT/G and CCNT/G substrates, from the maximal surface roughness to a lower one, the Ga droplet changes from the wetting state to the D state. Ga films on CNT/G surfaces exhibit a CB state when the surface roughness is large enough. Films on the CCNT/G surface with a higher roughness are in a CB or MW state, but the contact angle is quite close to a theoretical CB state. In addition, the stability of the MW state is greater than the CB state, so liquid Ga on the CCNT/G substrate achieves a D state with decreasing roughness later than does that on the CNT/G substrate. As for the CNC/G substrate, there are two transition states from the D state to the wetting state with decreasing roughness.

Quere etc. found that the CB state is more stable than the W state in thermodynamics when Young’s contact angle *θ_Y_* is larger than the critical angle *θ_C_* [46]:

(8) $$\;\theta_{Y}>\theta_{C},\;\theta_{C}=\left(f_{c}-1\right)/\left(r-f_{c}\right).\;$$

For the CNC nanopillar-patterned surfaces, liquid Ga wetting on graphene exhibited *θ_Y_* = 127.3°, while the surface roughness *f_c_* of 0.404, 0.223, 0.152, 0.095, 0.077, and 0.061 yield *θ_C_* = 97.9°, 105.87°, 112.1°, 120.5°, 122.2°, and 128.53°, respectively. This means that the W state (*f_c_* ≥ 0.077) is less stable than the CB state and the increasing *f_c_* of the substrate enhances the stability of the CB state. Therefore, liquid Ga is more easily dewetted on the CNC/G substrate by raising the *f_c_* and only experiences two wetting stages in the range of all the theoretically possible roughness. In a word, the stability order of these three wetting states is W < MW < CB. When *f_c_* ≤ 0.061, liquid Ga on carbon surface exhibits a thermodynamically stable W state but a fragile and unstable CB state.

**Figure 5:**
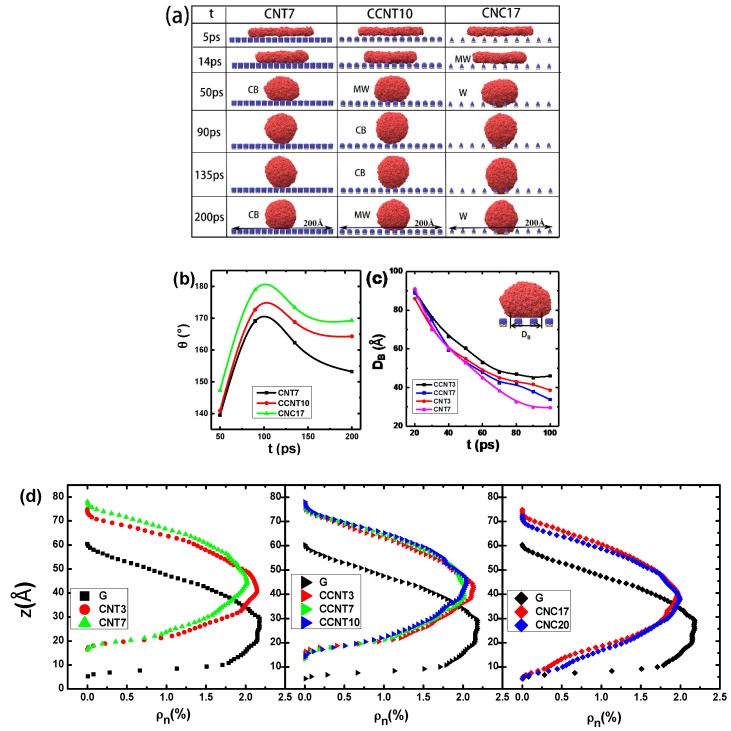
Morphological evolution of the liquid Ga films on three kinds of surfaces along the T line. (**a**) Snapshots of Ga droplets during the wetting process; (**b**) the contact angle of Ga droplet as a function of time, (**c**) the variation of the bottom diameter DB of the Ga droplet, and (**d**) the Ga number density *ρ_nz_* along the *z*-direction at 200 ps.

The snapshots in Figure 5a display the morphological evolution of the liquid Ga films on three kinds of surfaces along the T line, from the initial state to the stable state. Surface tension and weak interaction between the liquid film and substrate are the driving force for the movement and deformation of liquid films during the D process, dominating the initial horizontal movement and the upward flow of a subsequent droplet. Therefore, the Ga droplet firstly spreads over the substrate until its diameter reaches a maximum and then begins to shrink. In the case of CNT7/G substrates, the contact angles of the Ga droplet appear to show a similar trend, experiencing a CB state in the whole process, as shown in Figure 5a,b. The contact angle of the Ga droplet on CCNT10/G, from 50 to 200 ps, firstly rises and then reduces, which corresponds to the CB–W state and the MW state. Similarly, the contact angles of the Ga droplet on CNC17/G substrates at 50, 90, 135, and 200 ps are 147.3°, 178.9°, 173.4°, and 169.3°, respectively, corresponding to the change from the MW state to the W state. Figure 5c shows the variation of the bottom diameter of the Ga droplet on the CNT/G and CCNT/G substrates. The higher the surface friction is, the larger the occupied surface area is, and the smaller the contact angle is.

Consequently, we compared the density profiles of all the wetting states at 200 ps on both the rough and smooth surfaces, as shown in Figure 5d. It can be observed that the number densities of Ga drop close to the graphene change notably and slower than the rough surfaces. That is, the mass center of liquid Ga on the smooth surface is lower than that on the rough surface because of the smaller contact angle and good wettability.

**Figure 6:**
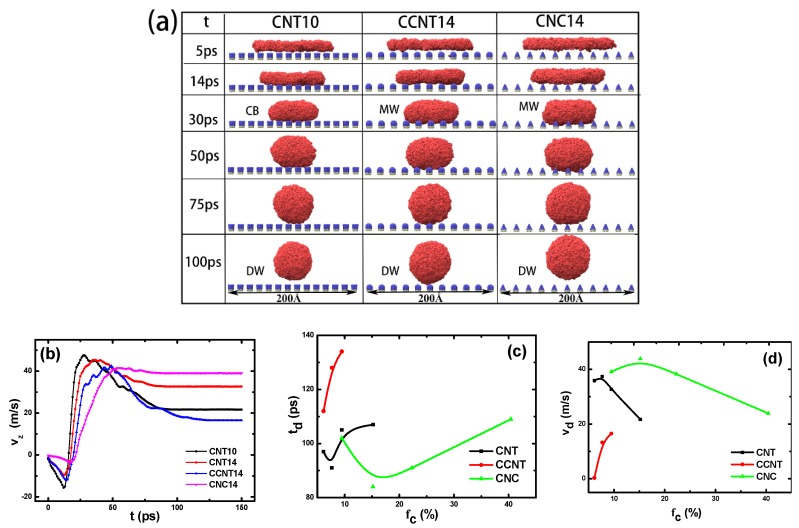
The detaching properties of liquid Ga at the D state. (**a**) Snapshots of Ga droplets during the dewetting process; (**b**) the temporal evolution of velocity *v_z_* of the Ga droplet in the *z*-direction; (**c**) the detachment time *t_d_*; and (**d**) the detachment velocity *v_d_* with *f_c_*.

Figure 6a presents snapshots of the liquid Ga films on the three different substrates at the D state. It was found that liquid Ga on the CNT14/G surface experiences variation from the CB state to the D state. However, on the CCNT14/G and CNC14/G surfaces, it undergoes the transition from the MW state to the D state. Compared with the CNT10/G substrate, it is more challenging to change the liquid Ga films to the D state, which suggests that the liquid in the MW state exhibits a more stable state. Figure 6b shows the velocity *v_z_* of the mass center of the Ga droplet in the *z*-direction. In the whole D process, the Ga droplet firstly falls off until the *v_z_* reaches zero and then rebounds with fluctuation to reach an equilibrium. In consideration of the initial upside-down Ga dish, the edge section of liquid film falls off more quickly than the center section. Figure 6c,d show the detachment time td and velocity *v_d_* versus the surface fraction *f_c_*. Their variation results from the conversion from the potential energy to the kinetic energy, which is affected by the *f_c_* and the morphology of nanopillars. For instance, as shown in Figure 6c, our simulation results demonstrate that the Ga droplet more easily rebounds from CNT/G and CNC/G substrates than it does from CCNT/G with the same *f_c_*. On the CNT/G substrate, the *v_d_* decreases with the increase of the *f_c_*, which implies that a large *f_c_* facilitates the conversion from the potential energy to the kinetic energy. Surprisingly, it was observed that the td decreases and then increases, but the *v_d_* varies inversely with the increasing *f_c_* on the CNT/G and CNC/G substrates, whereas, on the CCNT/G substrate, both td and *v_d_* augment with increasing *f_c_*.

### 3.4. Interaction Potentials and the Work of Adhesion

**Figure 7:**
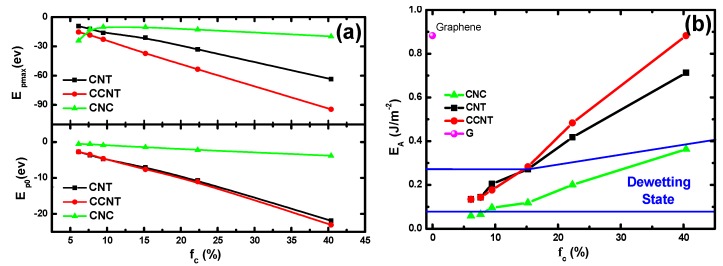
(**a**) The *E_pmax_* (the maximum interactions) and interactions *E_p_*_0_ (*t* = 0 ps) between Ga and C atoms of the associated substrate concerning the area fraction; and (**b**) the adhesion energy per unit area *E_A_* of each substrate with the area fraction increasing.

To further understand why the aforementioned transition occurs, we would like to discuss the energy efficiency, which has a close relation to the different surface topography thermodynamically. Figure 7a illustrates the directly proportional relationship between the roughness and the potential energy of three rough surfaces, which is relevant to the carbon atom numbers confined by the Lennard–Jones potential. For example, the CNC nanopillars containing the fewer atoms causes the whole substrate to have a smaller potential energy value compared with the other two substrates. This could explain why, initially, the upside-down Ga dish on the CNC3/G surface disappears more slowly, and the depositing velocity on the CNC3/G surface is slower than that on the CNT3/G and CCNT3/G. In addition, it suggests that, when the Ga droplet on CNT/G or CCNT/G change from the D state to the wetting state with increasing roughness, the adhesion also changes from a weak absorption to a strong one, and the surface finally loses its superhydrophobicity. However, only a slight difference in potential energy *E_p_*_0_ was found between the CNT/G and CCNT/G substrates, and the difference in maximal potential energy *E_pmax_* between the two surfaces was more evident. Herein, the *E_pmax_* is the maximum interaction, and *E_p_*_0_ is the interaction (*t* = 0 ps) between Ga and C atoms of the associated substrate. Looking back at Figure 3e–g, the diameter of the upside-down Ga dish is of little difference between CNT/G and CCNT/G substrates at 8 ps, but the more considerable potential energies make the dish on the CNT/G diminish and make the contact angle more significant. Notably, when liquid Ga on the CNC/G surface is situated at the wetting state, the *E_pmax_* is inversely proportional to the *f_c_* because of the existence of the CNC nanopillars. It could be concluded that the surface potential energy is affected mainly by the surface roughness and topography.

However, far and away, it is not overall and systematic to only discuss the interactions between Ga and C atoms. The free energy *G* is crucially important for exploring the Ga wettability. Basically, the adhesion energy per unit area can be calculated by the following equation [36]:

(9) $$\;E_{A}=\Delta GA^{-1}=\left(\Delta H-T\Delta S\right)A^{-1}.\;$$

The change in adhesion energy EA arises from two contributions. On the one hand, one part is called Δ*H_ls_.* When carbon substrates are turned into purely repulsive surfaces, there is interaction variation between liquid Ga and the associated substrate. On the other hand, the Ga–Ga interaction in the liquid Ga bottom structure near carbon substrates also changes. Indeed, it can be seen in Figure 5d that the carbon substrates induce the bottom of liquid Ga to form order layers. A contribution Δ*H_ll_* quantifies this effect on the liquid Ga structure. Therefore, the enthalpy change Δ*H* is the sum of two contributions, Δ*H =* Δ*H_ls_ +* Δ*H_ll_.* Figure 7b shows the adhesion energy of Ga films on CCNT/G, CNT/G, CNC/G, and graphene. It can be seen that the adhesion energy increases as *f_c_* increases, which means that higher adhesion energy values result in an improved wettability of the substrate. The transition diagram in Figure 7b provides a powerful tool to predict the wetting state of the liquid Ga film on various graphene-based substrates.

Herein, the relationship between *f_c_* and *E_A_* demonstrates that the adhesion energy not only well determines the wettability trends of the surfaces but also supplements to explain the influence of the top morphology of nanopillars. It is clear that the shape of Ga droplets on CNT/G and CCNT/G surfaces is almost the same with the same *f_c_* and their *E_A_* values are similar regardless of the wetting or D state. However, on account of the little difference in the top morphology of nanopillars, there are many concerns regarding detachment time td (as displayed in Figure 5c), detachment velocity *v_d_* (as shown in Figure 5d), contact angle, the maximum of potential energy *E_pmax_*, and even the wetting pattern transitions with the decrease in *f_c_*. According to the relationship between contact angle and adhesion energy (seen in Figure 5d and Figure 7b), a small contact angle corresponds well to a high adhesion energy. By its very nature, the pattern of the nano-decoration on the graphene surface exerts a profound impact on the structural evolution of the liquid Ga film on the substrate and presents a novel method by which the liquid wettability can be controlled.

## 4. Conclusions

In summary, we investigated the wetting states of liquid Ga films on three kinds of nano-patterned carbon surfaces via MD simulations. Surface wettability can be easily tuned not only among the typical wetting states such as the CB state, the MW state, and the W state but also between wetting and the D state by modifying the nanopattern on graphene. The adsorption strength of liquid on patterned substrates, compared with the smooth surface, is decreased not only due to the diminished surface energy along with dwindling roughness but also because of the upside-down Ga dish in the initial stage, which both reduce the contact between Ga atoms and substrate. Our results reveal that the wettability of liquid on the solid surface is determined concurrently by their surface energy and surface geometrical microstructure. The top morphology of nanopillars affects the wetting transition of liquid Ga by not only lessening the interaction between liquid and solid but also changing the movement pattern of liquid at the beginning. The work of adhesion will be appropriately used to predict the wettability of drops from both smooth and rough surfaces. This work improves our understanding of wetting transitions and is expected to better facilitate the superhydrophobic development surfaces, such as self-cleaning nano-materials and the design of stretchable electronic devices.

## Figures and Tables

**Table 1:** Simulation results of wetting states on different substrates.

*S* (Å)	*r* ^a^	*f_c_* ^a^	CNT/G ^b^			CCNT/G			CNC/G		
			*θ_CA_*	*θ_TCA_* ^c^	Model ^d^	*θ_CA_*	*θ_TCA_*	Model	*θ_CA_*	*θ_TCA_*	Model
3.4	4.743	0.404	138.3°	147.2°	CB	134.8°	147.2°	CB	180°	180°	D
7	3.064	0.223	153.2°	155.8°	CB	146.9°	62.5°	MW	180°	180°	D
10.5	2.404	0.152	180°	180°	D	164.3°	59.7°	MW	180°	180°	D
14	1.877	0.0948	180°	180°	D	180°	180°	D	180°	180°	D
17.5	1.710	0.0767	180°	180°	D	180°	180°	D	169.3°	180°	W
20	1.568	0.0614	180°	180°	D	180°	180°	D	160.1°	161.4°	W

^a^*r* and *f_c_* are the roughness ratio and the area fraction of the substrate respectively; ^b^ G is the abbreviation of graphene; ^c^ *θ_TCA_* is the theoretical contact angle derived from Equations (1)–(3); ^d^ the models are the Wenzel’s model (W), the Mixed Wetting model (MW), the Cassie–Baxter model (CB), and the dewetting model (D), respectively.

## Nomenclature

CNT	Carbon nanotube
CCNT	Caped Carbon nanotube
CNC	Carbon nanocone
CB state	Cassie–Baxter state
W state	Wenzel state
MW state	Mixed wetting state
D state	Dewetting state
*θ_CA_*	contact angle
*θ_Y_*	Young’s contact angle
*θ_TCA_*	theoretical contact angle
*θ_C_*	critical angle
*R*	botom radius of a nanopillar
*S*	distance between any two nearest neighbor nanopillars
*a*	height of a nanopillar
*L*	length side of rough substrate
*n*	numbers of the nanopillarss
*r*	roughness ratio of substrate
*f_c_*	area fraction
*E_A_*	adhesion energy per unit area
*ε*	well depth
*σ*	size parameter
*ρ_ni_*	number density
*E_pmax_*	the maximal potential energy
*E*_*p*0_	potential energy at 0 ps
*G*	free energy
*H*	enthalpy
